# Buccal cheek mucosa solitary fibrous tumor. Case report

**DOI:** 10.4317/jced.61157

**Published:** 2023-12-01

**Authors:** Alba García-López-Chicharro, Marta-María Pampín-Martínez, Clara López-Martínez, Íñigo Aragón-Niño, José-Luis Cebrián-Carretero

**Affiliations:** 1 Oral and Maxillofacial Surgery Department. Hospital Universitario La Paz, Madrid, España

## Abstract

Solitary fibrous tumor (STF) is a mesenchymal tumor that mainly appears in the pleura. Its presence in the oral cavity is very uncommon, being the buccal mucosa the most frequent location. Imaging cannot distinguish this entity between other types of tumors, being histological and immunohistochemical studies essential for its diagnosis. Immunohistochemical stains typically show positive results for CD34, Bcl2, and CD99. Surgical removal with wide margins is the gold standard treatment, requiring a close follow up due to recurrence risk. We present a case report of a solitary fibrous tumor located in the buccal cheek mucosa and the surgical approach.

** Key words:**Solitary fibrous tumor, Buccal mucosa, Intraoral, Immunohistochemical markers.

## Introduction

Solitary fibrous tumor is a tumor of mesenchymal origin that was initially characterized by Klemperer and Rabin as a pleural tumor (1). It is also known as “localized fibrous mesothelioma”, “solitary fibrous mesothelioma” and “submesothelial fibroma”. While it mainly appears in the pleura, it has also been documented in other locations such as the abdominal cavity, upper respiratory tract, and soft tissue. Nevertheless, its presence in the oral cavity is exceedingly uncommon, with only a few reported cases.

Immunofluorescence positivity to CD34, CD99 and negativity to Bcl-2 markers helps with the diagnosis (2). It has been recently described a recurrent chromosomal translocation affecting NAB2 and STAT6 genes in this kind of tumors (3). Surgical removal remains the gold standard for its treatment. In this article we present a case report of a solitary fibrous tumor located in the buccal mucosa and the surgical approach.

Case Report

A 61-year-old male presented with a left buccal mucosa tumor initially diagnosed as lipoma by ultrasound imaging that was present for two years. The patient did not report constitutional syndrome, being his unique concern the discomfort due to occasional biting of the buccal mucosa. Due to progressive growth, fine needle aspiration (FNA) was performed, where moderate aggressivity spindle cell tumor images were found. Immunohistochemical study was limited due to the low cellularity obtained (CD34+, actine and smooth muscle negative, SOX10-, STAT6-).

**Figure 1:**
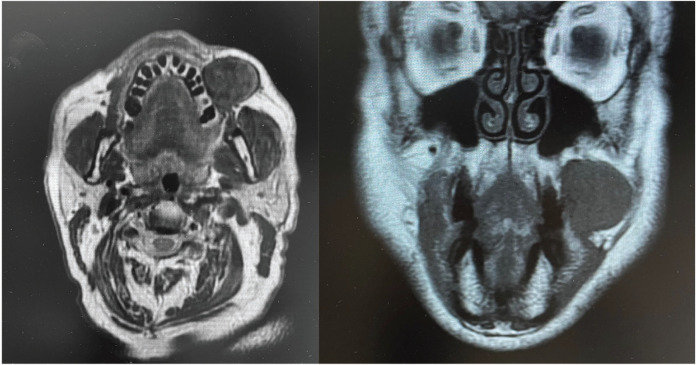
MRI images showing a well-defined mass in the buccal space.

MRI images revealed an isointense in T1-weighted sequences mass, localized in the buccal space, with non-aggressive appearance, well-defined borders of 33x31x27mm size. Hypointense in T2 and Stir-weighted sequences (Fig. 1). It was closely related with buccinator muscle. It didn’t exhibit suggestive lipoma features, although they recommended anatomopathological study because a low grade liposarcoma couldn’t be ruled out. Differential diagnosis was a low grade liposarcoma, a neurogenic origin tumor or a solitary fibrous tumor. PET-TC ruled out distant metastasis and locoregional lymphadenopathies, showing moderate activity in the tumor location.

**Figure 2:**
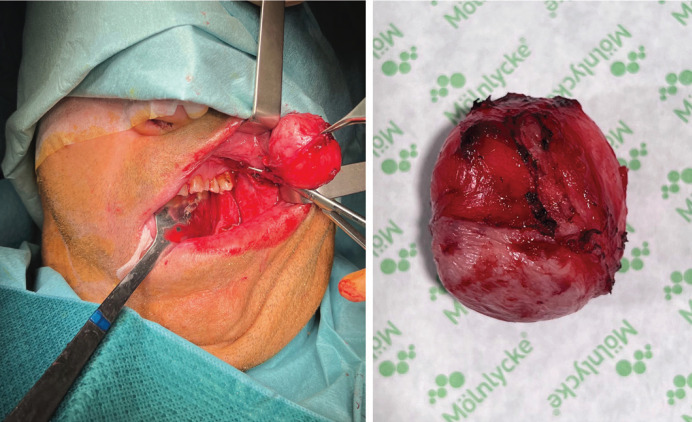
Surgical excision. Tumor.

**Figure 3:**
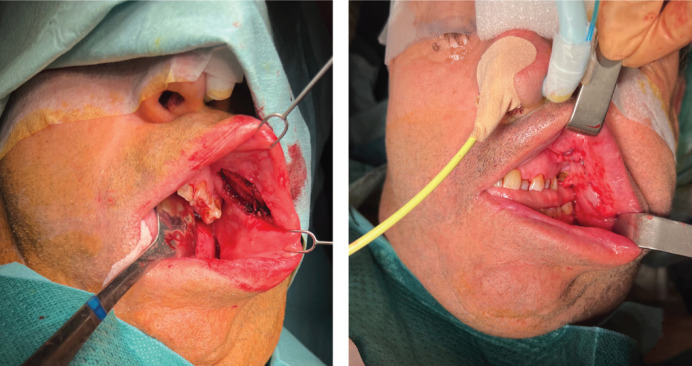
Surgical reconstruction using buccal fat flap. Final result.

Surgery was the chosen treatment and surgical excision and reconstruction with buccal fat flap was performed under general anesthesia (Figs. 2,3).

Postoperative anatomopathological diagnosis was a 2.3cm diameter low grade solitary fibrous tumor. Non tumoral necrosis, nuclear atypia or mitosis was found. Margins were tumor-free. Immunohistochemical study revealed the following: CD34 positive, STAT6 positive. S100, SOX10, CKAE1/AE3, CD31, desmin and AML were negative. Ki67 was 3%.

Patient’s postoperative recovery was good, being discharged after a 5-day hospital stay with a nasogastric tube for surgical wound hygiene reasons.

Case was presented in our head and neck oncology committee, where it was concluded that no adjuvant treatment was necessary. Follow up of the patient has not shown recurrence signs during a 2 year follow up.

## Discussion

Solitary fibrous tumor (SFT) is a very uncommon intraoral tumor. It mainly occurs in the pleura. Cox *et al*. described 153 head and neck solitary fibrous tumors (4). 26.1% developed in the buccal mucosa, 9.2% in the nasal, 7.2% in the tongue and 6.5% in the orbit. The rest was found in the pharynx. Buccal mucosa is most commonly affected by the benign variant and the tongue by the malignant variant. Fernanda Bispo *et al*. reviewed a total of 74 publications reporting a total of 150 STF. 90% of SFT were benign, whilst only 14 cases were malignant (5).

Clinically, SFT in the oral cavity typically presents as a well-defined submucosal, asymptomatic, and slow growing mass. It can often be mistaken for other conditions such as mucocele, salivary gland tumors, lipoma, vascular malformationsand leiomyoma, among others (6).

SFT can be identified using different imaging techniques such as computed tomography (CT), magnetic resonance imaging (MRI), and positron emission tomography-CT (PET-CT). It’s essential to distinguish these lesions from neurofibromas, schwannomas, leiomyomas, and hemangiopericytomas. To differentiate between them, pathologists employ hematoxylin and eosin (H&E) staining along with immunofluorescence staining.

Microscopic features that might suggest the presence of SFT include well-defined growth of spindle-shaped cells within a stroma that can vary in vascularity and collagen content. Identifying staghorn-shaped vessels within the lesion can be a valuable indicator, and the immunohistochemical stains that confirm the diagnosis typically show positive results for CD34, Bcl2, and CD99 (2). More recently, the use of STAT6 staining has been demonstrated as a sensitive and specific marker for SFT (2,3).

Typically, the preferred approach involves surgical removal with a wide excision margin, and additional treatment is often unnecessary, particularly when a low-grade STF is present. This is especially true for SFTs located in the head and neck region, as they seldom recur or metastasize.

In the study conducted by Demicco *et al*. 110 cases of whole-body SFTs were documented (7). Among these, 103 cases underwent surgical treatment, and within this group, 15% of the patients also received additional therapy, such as radiotherapy and/or chemotherapy. After a median follow-up period of 48 months, a significant proportion of patients, specifically 29%, experienced local recurrence and/or metastasis.

In general, SFTs tend to follow a benign clinical course, but their behavior can be unpredictable, and there is often a limited correlation between their appearance and their clinical behavior. That’s why it’s important to emphasize the need for vigilant long-term monitoring even after complete removal (5,8,9).

In this case, the tumor, despite being big in size, was well circumscribed and limited and surgery went uneventfully. A low-grade STF was demonstrated upon histological examination and therefore no adjuvant treatment was needed in this patient, who is presently free of recurrence after 2 years of follow up.

In conclusion, SFT tumors are very uncommon in the head and neck area. When located in this area, buccal mucosa is the most common apparition site. Differential diagnosis must be done with other entities such as lipomas, neurogenic origin tumors, liposarcoma and leiomyomas. Imaging studies reveal a well-circumscribed tumor but cannot distinguish another kind of tumors, that’s why anatomopathological and immunohistochemical studies are essential for a precise diagnosis. Surgical excision remains the gold standard for STF treatment being very important to have a close follow-up with the patient due to the high risk or recurrence and distant metastasis in the malignant varieties.
